# Clinical and economic burden of surgical site infections in inpatient care in Germany: A retrospective, cross-sectional analysis from 79 hospitals

**DOI:** 10.1371/journal.pone.0275970

**Published:** 2022-12-28

**Authors:** Christian Eckmann, Axel Kramer, Ojan Assadian, Steffen Flessa, Claudia Huebner, Kristian Michnacs, Christian Muehlendyck, Kim Mirjam Podolski, Michael Wilke, Wolfgang Heinlein, David John Leaper

**Affiliations:** 1 Department of General, Visceral and Thoracic Surgery, Klinikum Hann. Muenden Academic Hospital of Goettingen University, Hann. Muenden, Germany; 2 Institute of Hygiene and Environmental Medicine, University Medicine Greifswald, Greifswald, Germany; 3 Regional Hospital Wiener Neustadt, Wiener Neustadt, Austria; 4 School of Human & Health Sciences, University of Huddersfield, Huddersfield, United Kingdom; 5 Department of Health Care Management, Faculty of Law and Economics, University of Greifswald, Greifswald, Germany; 6 Johnson & Johnson Medical GmbH, Norderstedt, Germany; 7 Inspiring-health GmbH, Munich, Germany; 8 University of Newcastle upon Tyne, Newcastle upon Tyne, United Kingdom; URCEco Ile de France Hopital de l’Hotel Dieu, FRANCE

## Abstract

**Background:**

Surgical site infections (SSI) present a substantial burden to patients and healthcare systems. This study aimed to elucidate the prevalence of SSIs in German hospitals and to quantify their clinical and economic burden based on German hospital reimbursement data (G-DRG).

**Methods:**

This retrospective, cross-sectional study used a 2010–2016 G-DRG dataset to determine the prevalence of SSIs in hospital, using ICD-10-GM codes, after surgical procedures. The captured economic and clinical outcomes were used to quantify and compare resource use, reimbursement and clinical parameters for patients who had or did not have an SSI.

**Findings:**

Of the 4,830,083 patients from 79 hospitals, 221,113 were eligible. The overall SSI prevalence for the study period was 4.9%. After propensity-score matching, procedure type, immunosuppression and BMI ≥30 were found to significantly affect the risk of SSI (p<0.001). Mortality and length of stay (LOS) were significantly higher in patients who had an SSI (mortality: 9.3% compared with 4.5% [p<0.001]; LOS (median [interquartile range, IQR]): 28 [27] days compared with 12 [8] days [p<0.001]). Case costs were significantly higher for the SSI group (median [IQR]) €19,008 [25,162] compared with € 9,040 [7,376] [p<0.001]). A median underfunding of SSI was identified at €1,534 per patient.

**Interpretation:**

The dataset offers robust information about the “real-world” clinical and economic burden of SSI in hospitals in Germany. The significantly increased mortality of patients with SSI, and their underfunding, calls for a maximization of efforts to prevent SSI through the use of evidence-based SSI-reduction care bundles.

## Introduction

Surgical site infections (SSI) are among the most common healthcare-acquired infections (HAIs), with rates varying following the type of surgery [1]. European surveillance data shows that the rate of SSI between 2008 and 2016 has not been reduced in all major surgical subpopulations [2] and related to the capture of current surveillance data, it is likely that the actual number of SSIs is underestimated.

SSIs increase postoperative mortality and morbidity, resulting in increased length of stay (LOS), further surgical procedures related to SSIs, and an increased need for post-operative intensive care [1, 3–6]. Consequently, SSIs increase the financial burden of surgical procedures, leading to a significant impact on national health expenditure.

The number of surgical procedures undertaken in Germany has increased over the last decade, averaging at 15.9 million per year during the 2010–2016 period [7]. According to the latest European Centre for Disease Prevention and Control (ECDC) point prevalence data, Germany has one of the lowest rates of SSI [2]. The overall prevalence of HAI in Germany in 2016 was reported to be 4.6%, with SSI representing 22.4% of reported HAIs (an overall prevalence of 1.08%) based on data from 64,412 patients in 218 participating hospitals [8]. The individual SSI rates for German hospitals are published on the National Reference Centre System for Surveillance of Nosocomial Infections (Krankenhaus-Infektions-Surveillance-System; KISS) website [9].

Reporting of SSI data in Germany is conducted through the Hospital Infection Surveillance System with focus on SSI (OP-KISS) at the National Reference Center for Surveillance of Nosocomial Infections (NRZ) [9]. This is based on the US surveillance-systems of the National Healthcare Safety Network (NHSN) from the Centre for Disease Control and Prevention (CDC). Since January 2017 the CDC definitions were replaced by local definitions of the NRZ [10], although these new definitions are based on those of the CDC. The NRZ provides participating hospitals with their own electronic system for documenting data. The hospitals which participate in the data collection must first take an introductory course in which the method of surveillance is taught, and the diagnosis of SSI additionally explained by using sample illustrative cases of SSIs. The comparability of the data across centers is therefore not guaranteed, and the quality and validity of the data collection is inherently subject to human error or omission. Based on these circumstances, it can be expected that the inpatient SSI may be under-reported in individual hospitals or individual departments of the reporting hospitals. Additionally, actual SSI rates are likely to be higher after discharge due to incomplete or missing SSI documentation.

The considerable economic impact of SSI has been established in several European countries and the US [3, 4, 11], however, no economic analysis has been conducted to establish the direct costs associated with SSI in German hospitals based on standardized “real-world” data. The present study was designed to assess the clinical and economic burden of SSI in German hospitals using prospectively collected routine data which was captured as part of the annual German diagnosis-related group (G-DRG) hospital resource use and reimbursement scheme.

## Methods

### Study design and setting

This study was a retrospective, cross-sectional, multicenter database study designed to assess the prevalence of SSI in a representative sample of German hospitals, and to compare LOS as well as costs for patients that experienced an SSI, compared with those who did not, over the period 2010–2016.

### Data source

The G-DRG system is a case-based “lump sum” and procedural rate system and is used within Germany to determine reimbursement to hospitals following inpatient stays. In order to do this, specific data are captured at patient discharge as mandated by the Hospital Remuneration Act (Krankenhausentgeltgesetz, KHEntgG) §21 [12]. The dataset contains extensive but no patient identifying anonymous data, including age, gender, admission date, discharge date, hours of mechanical ventilation, costs, and more administrative information. Importantly, the dataset also captures procedures (via OPS-301 code) and diagnoses (via ICD-10-GM code) for each hospital stay [13]. In the §21 dataset diagnoses, procedures, case data and billing information are mandatory for all reporting-hospitals, cost data are voluntarily reported by predefined reporting-hospitals [13].

Diagnoses are divided into two categories in the G-DRG system: principle diagnosis (PDx) and secondary diagnosis (SDx). The PDx ICD-10-GM code reflects the reason for admission, and the SDx can be up to 50 ICD-10-GM codes which provide additional information regarding each patient’s condition. ICD-10-GM codes are associated with resource use within the system. DRGs are calculated based on cost data, per case, collected from the participating reporting-hospitals for staff, drugs, medical disposables, infrastructure, material costs, and LOS [14].

In 2010, there were 316 hospitals and in 2016, there were 340 hospitals in Germany which were nominated to deliver the fully anonymized dataset including their cost data to the German Institute for Reimbursement in Inpatient Care (Institut für das Entgeltsystem im Krankenhaus, InEK) [13].

For this study a subset of this dataset has been used. It was made available by the German Scientific Society of Gastroenterology, Digestive and Metabolic Diseases (Deutsche Gesellschaft für Gastroenterologie, Verdauungs- und Stoffwechselkrankheiten; DGVS) for research purposes. The DGVS is responsible for the collection and management of this subset of data from 79 specific hospitals. These data have been used for several published clinical and economic studies to date [15, 16]. The distribution of hospitals is similar to the total German distribution with a slight bias towards university and major urban hospitals [17].

### Study population

The study population included the hospital performance data of all patients in the DGVS data set of 79 hospitals which had undergone pre-defined surgical procedures, as captured by German OPS-301 codes. Surgical procedures of interest broadly covered cardiac, colorectal, gynecological, spinal, thoracic, upper gastrointestinal, orthopedic and trauma specialties; the complete list of procedures of interest can be found in S1 Table. The population was then separated into two groups based on whether they had experienced an SSI during their hospital stay or did not (defined as ‘SSI’ and ‘non-SSI’ groups), based on the presence of a pre-defined German modification of the International Statistical Classification of Diseases and Related Health Problems (ICD-10-GM) code (Table 1).

**Table 1:** ICD-10-GM codes for the identification of SSI and designated NRZ classification.

Code	Description	NRZ classifier
T81.4	Infection following a procedure not elsewhere classified	A1/A2
T82.6	Infection and inflammatory reaction due to cardiac valve prosthesis	A3
T82.7	Infection and inflammatory reaction due to other cardiac and vascular devices, implants and grafts	A3
T84.5	Infection and inflammatory reaction due to internal joint prosthesis	A3
T84.6	Infection and inflammatory reaction due to internal fixation device [any site]	A3
T84.7	Infection and inflammatory reaction due to other internal orthopedic prosthetic devices, implants and grafts	A3

Abbreviations: NRZ, Nationales Referenzzentrum

Each ICD-10-GM code was then mapped to the NRZ classification within the framework of the systemic schemes: A1/A2 (superficial incisional/deep incisional SSI) or A3 (organ and body cavity/ pace SSI) (Table 1) [10]. A1/A2 was assigned to ICD-10-GM code T81.4 and A3 was assigned to all other ICD-10 GM codes that indicate infections following procedures (Table 2). Since the code for peritonitis and device-affected infection (joint prosthesis, cardiac and vascular devices) does not differentiate between those present on admission and those developed postoperatively, this was captured as an outcome parameter in the baseline characteristics (Table 3). The logic of the ICD-10 coding identifies some cases of peritonitis, joint prosthesis infection (PJI), valve prosthetic endocarditis (PVE) and vascular devices as cases of SSI which can be assigned to A3 to follow the A1-A3 classification. As pre- and post-operative peritonitis is complex to differentiate based on coded data, we did not include it as a SSI.

**Table 2:** Frequency of SSI by procedure type and NRZ classification, 2010–2016.

Procedure type	Procedures, n (%)	SSI, n (%)	A1/A2 SSI, n (%)	A3 SSI, n (%)
All	221,113 (100·0)	10,807 (4·9)	5,997 (2·7)	4,810 (2·2)
Cardiac Surgery	39,676 (17·9)	2,109 (5·3)	1,215 (3·1)	894 (2·3)
Thoracic Surgery	9,288 (4·2)	202 (2·2)	121 (1·3)	81 (0·9)
Wedge resection, VATS	3,427 (1·5)	39 (1·1)	23 (0·7)	16 (0·5)
Wedge resection, open	1,970 (0·9)	64 (3·2)	40 (2·0)	24 (1·2)
Other	3,891 (1·8)	99 (2·5)	58 (1·5)	41 (1·1)
Small bowel resection	7,465 (3·4)	1,030 (13·8)	818 (11·0)	212 (2·8)
Open	6,955 (3·1)	1,005 (14·5)	799 (11·5)	206 (3·0)
Lap	510 (0·2)	25 (4·9)	19 (3·7)	6 (1·2)
Colon resection	26,249 (11·9)	2,230 (8·5)	1,906 (7·3)	324 (1·2)
Hemicolectomy, right/open	6,939 (3·1)	587 (8·5)	481 (6·9)	106 (1·5)
Hemicolectomy, right/lap	1,571 (0·7)	71 (4·5)	65 (4·1)	6 (0·4)
Sigma resection, open	3,184 (1·4)	417 (13·1)	354 (11·1)	63 (2·0)
Sigma resection, lap	4,912 (2·2)	173 (3·5)	164 (3·3)	9 (0·2)
Other	9,643 (4·4)	982 (10·2)	842 (8·7)	140 (1·5)
Rectal resection	11,502 (5·2)	937 (8·1)	818 (7·1)	119 (1·0)
Deep anterior resection, open	2,561 (1·2)	221 (8·6)	191 (7·5)	30 (1·2)
Deep anterior resection, lap	1,689 (0·8)	68 (4·0)	55 (3·3)	13 (0·8)
Other	7,252 (3·3)	648 (8·9)	572 (7·9)	76 (1·0)
Gynecology	26,416 (11·9)	359 (1·4)	329 (1·2)	30 (0·1)
Trauma	4,145 (1·9)	149 (3·6)	77 (1·9)	72 (1·7)
Hip endoprosthesis	48,493 (21·9)	1505 (3·1)	295 (0·6)	1,210 (2·5)
Primary, open	44,859 (20·3)	861 (1·9)	240 (0·5)	621 (1·4)
Revision/replacement/removal, open	3,634 (1·6)	644 (17·7)	55 (1·5)	589 (16·2)
Knee endoprosthesis	27,198 (12·3)	1,791 (6·6)	108 (0·4)	1,683 (6·2)
Primary, open	23,877 (10·8)	531 (2·2)	76 (0·3)	455 (1·9)
Revision/replacement/removal, open	3,321 (1·5)	1,260 (37·9)	32 (1·0)	1,228 (37·0)
Spine surgery	20,681 (9·4)	495 (2·4)	310 (1·5)	185 (0·9)

SSI, surgical site infection; VATS, video-assisted thoracic surgery

**Table 3:** Comparison of baseline characteristics and outcomes between SSI and non-SSI cohorts, 2010–2016.

	Unadjusted			Adjusted		
	SSI	Non-SSI	P value	SSI	Non-SSI	P value
Cases, n (%)	10,807 (4·9)	210,306 (95·1)	··	10,804 (25·0)	32,393 (75·0)	··
A1/A2 SSI, n (%)	5,997 (55·5)	··	··	5,997 (55·5)	··	··
A3 SSI, n (%)	4,810 (44·5)	··	··	4,807 (44·5)	··	··
**Baseline characteristics**						
Male/female*, n (%)/,n (%)	6,004 (55·6)/4,795 (44·4)*	91,911 (43·7)/118,356 (56·3)*	<0·001	6,004 (55·6)/4,795 (44·4)†	18,207 (56·2)/14,166 (43·7)†	0·428
Age, years (95% KI)	67·4 (67·13–67·66)	66·0 (65·98–66·10)	<0·001	67·4 (67·13–67·66)	67·4 (67·22–67·52)	0·862
Primary diagnosis (eight most frequent), ICD-10 chapter						
Infections and parasitic diseases, 1, n (%)	128 (1·2)	554 (0·3)	<0·001	128 (1·2)	119 (0·4)	<0·001
Neoplasms, 2, n (%)	2,345 (21·7)	44,625 (21·2)		2,343 (21·7)	8,216 (25·4)	
Circulatory system, 9, n (%)	2,107 (19·5)	37,938 (18·0)		2,107 (19·5)	7,737 (23·9)	
Respiratory system, 10, n (%)	85 (0·8)	2,247 (1·1)		85 (0·8)	348 (1·1)	
Digestive system, 11, n (%)	1,812 (16·8)	18,019 (8·6)		1,812 (16·8)	2,555 (7·9)	
Musculoskeletal system, 13, n (%)	609 (5·6)	64,799 (30·8)		609 (5·6)	8,371 (25·8)	
Genitourinary system, 14, n (%)	170 (1·6)	10,934 (5·2)		170 (1·6)	746 (2·3)	
Injury, poisoning and certain other consequences of external causes, 19, n (%)	3,404 (31·5)	29,346 (14·0)		3,403 (31·5)	4,002 (12·4)	
Comorbidities						
Median CCI score (IQR)	2 (4)	1 (2)	··	2 (4)	2 (3)	··
Diabetes mellitus, n (%)	2,998 (27·8)	35,731 (17·0)	<0·001	2,996 (27·7)	8,796 (27·2)	0·244
Immunosuppression, n (%)	365 (3·4)	1,957 (0·9)	<0·001	364 (3·4)	874 (2·7)	<0·001
BMI ≥30 kg/m^2^, n (%)	1,805 (16·7)	26,843 (12·8)	<0·001	1,804 (16·7)	5,473 (16·9)	0·634
**Procedure type**						
Small bowel resection, n (%)	1,030 (9·5)	6,435 (3·1)	<0·001	1,030 (9·5)	1,257 (3·9)	<0·001
Cardiac surgery, n (%)	2,109 (19·5)	37,567 (17·9)		2,109 (19·5)	7,621 (23·5)	
Colorectal resection, n (%)	3,167 (29·3)	34,584 (16·4)		3,167 (29·3)	6,396 (19·7)	
Gynaecology, n (%)	359 (3·3)	26,057 (12·4)		359 (3·3)	2,139 (6·6)	
Hip & Knee endoprothesis, n (%)	3,296 (30·5)	72,395 (34·4)		3,295 (30·5)	9,748 (30·1)	
Spine surgery, n (%)	495 (4·6)	20,186 (9·6)		493 (4·6)	2,970 (9·2)	
Thoracic surgery, n (%)	202 (1·9)	9,086 (4·3)		202 (1·9)	1,836 (5·7)	
Trauma, n (%)	149 (1·4)	3,996 (1·9)		149 (1·4)	426 (1·3)	
**Outcomes**						
Median overall LOS, days (IQR)	28 (27)	11 (7)	<0·001	28 (27)	12 (8)	<0·001
Median pre-surgical LOS, days (±IQR)	2 (7)	1 (1)	<0·001	2 (7)	1 (2)	<0·001
Sepsis, n (%)	1,909 (17·7)	4,325 (2·1)	<0·001	1,909 (17·7)	1,067 (3·3)	<0·001
Mortality, n (%)	1,007 (9·3%)	6,039 (2·9%)	<0·001	1,007 (9·3%)	1,467 (4·5%)	<0·001
ICU stays, n (%)	5,962 (55·2%)	48,854 (23·2%)	<0·001	5,959 (55·2%)	10,022 (30·9%)	<0·001
Median case cost, € (IQR)	19,010 (25,190)	7,693 (6,118)	<0·001	19,008 (25,162)	9,040 (7,376)	<0·001
Median cost, ward, € (IQR)	5,107 (5,765)	2,395 (1,633)	<0·001	5,107 (5,763)	2,566 (1,869)	<0·001
Median cost, ICU, € (IQR)	2,041 (10,622)	0 (1247)	<0·001	2,041 (10,622)	568 (2,050)	<0·001
Median cost, OR, € (IQR)	4,565 (4,618)	2,854 (2,106)	<0·001	4,564 (4,613)	3,059 (2,366)	<0·001
Median G-DRG reimbursement, € (IQR)	15,086 (20,674)	7,869 (5,357)	<0·001	15,084 (20,661)	9,689 (6,414)	<0·001
Median contribution margin, € (IQR)	-1,537 (8,697)	596 (2,810)	<0·001	-1,534 (8,688)	633 (3,400)	<0·001

BMI, body mass index; CCI, Charlson Comorbidity Index; G-DRG, German diagnosis-related group; GI, gastrointestinal; ICD-10, International Statistical Classification of Diseases and Related Health Problems-10; ICU, intensive care unit; LOS, length of stay; OR, operating room; SD, standard deviation

*, 47 Patients undefined; †, 25 Patients undefined

**Fig 1:**
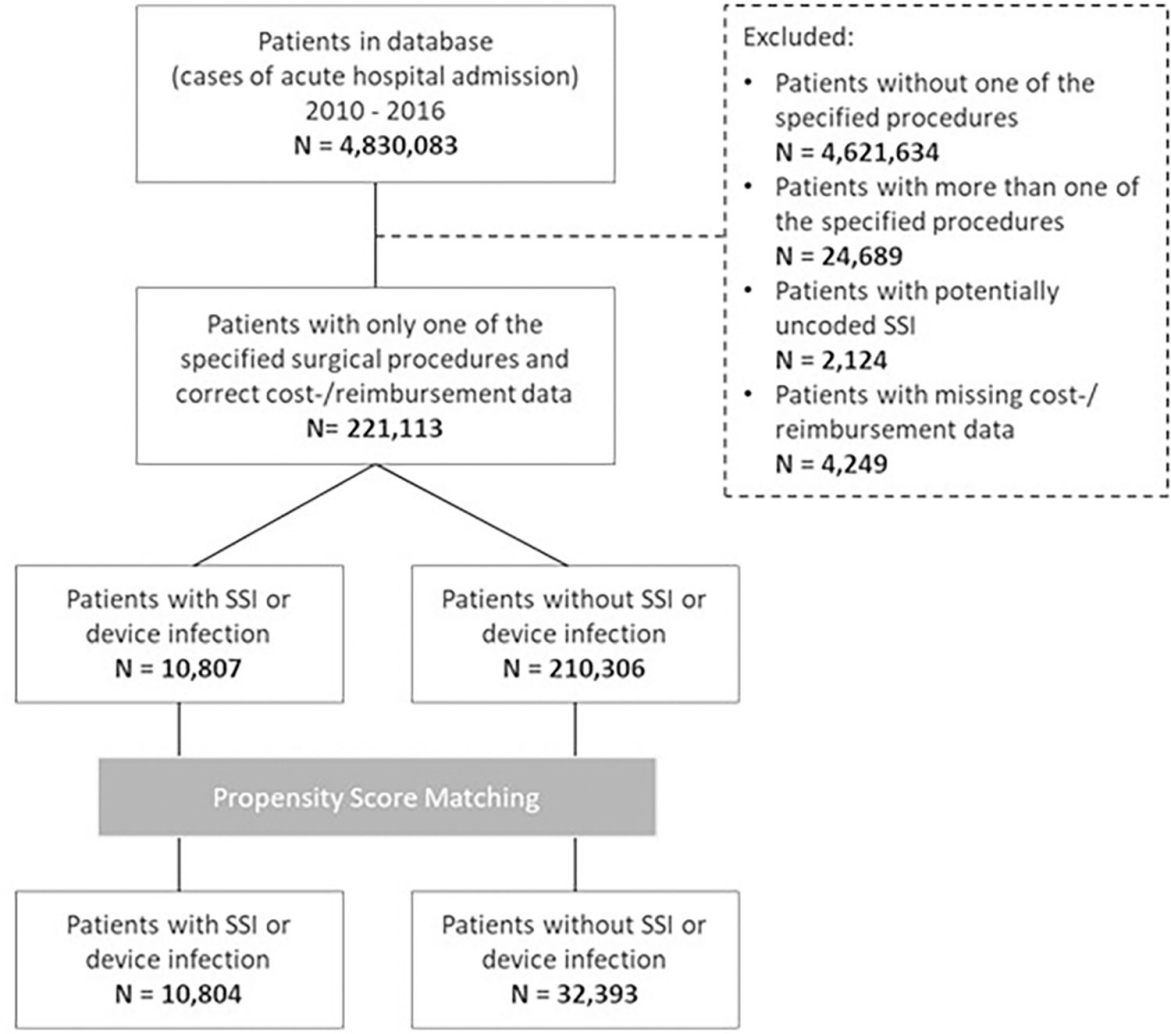
Flow diagram for case numbers, 2010–2016.

Patient reports were excluded if: i) cost or reimbursement data were missing; ii) the patient had more than one surgical procedure causal of the admission diagnosis undertaken during their billed hospital stay; and iii) the patient did not have an ICD-10-GM code for SSI despite the presence of an OPS-301 code that would suggest the presence of SSI (Fig 1).

These cases were determined according to 4,830,083 inpatient case reports captured in the database from 79 hospitals by the DGVS covering the years 2010–2016. After exclusion of ineligible cases according to the index procedures, 221,113 remained for analysis (Fig 1).

### Covariates

The following covariates were analyzed: age, sex, diabetes (differentiated according to type), BMI ≥ 30 kg/m^2^, immunosuppression, peritonitis, sepsis and the Charlson Comorbidity Index (CCI).

### Outcomes

The primary outcome was the period prevalence of SSI in the overall population. SSI cases were then stratified by procedure type and NRZ classifier.

Secondary outcomes were mortality, median overall LOS, median pre-surgical LOS and median case cost for the SSI and non-SSI groups. For three cost centers detailed analyses were undertaken: general ward, intensive care unit (ICU) and operating room.

Further outcomes of interest were total ICU stays, median G-DRG reimbursement and median contribution margin (reimbursement per case minus costs per case).

Baseline characteristics and outcomes for included patients were extracted and compared between SSI and non-SSI groups for both adjusted and un-adjusted analyses.

### Bias

Propensity score matching (PSM) was performed on the SSI and non-SSI groups to minimize bias from possible confounding variables.

### Quantitative variables and statistical methods

In a subgroup analysis of the overall study population (all patients undergoing at least one defined procedure), absolute values for SSI prevalence by procedure type and NRZ classifier were analyzed, without any statistical adjustment or assessment.

The analysis of categorical variables was performed using Chi-Square test (*x*^2^) test. For normal distributed mean values with continuous variables and standard deviations, the statistical calculation was carried out with the Student’s t-test. For non-normal distributed medians and IQR, the statistical calculation was carried out with the Mann-Whitney-U test. Statistical significance was defined as p<0.05. All tests were two-tailed.

To attempt to correct for possible confounders within this large sample size, PSM was conducted using a 1:3 (SSI: non-SSI) relation with the nearest-neighbour algorithm [18]. An additionally performed 1:1 matching (S2 Table) corrected the imbalance of adjusted data for immunosuppression and some operative procedures, but did not change anything regarding the statistical differences for the outcomes and was re-assuring the 1:3 matching approach. The propensity score was estimated using a logistic regression model in which SSI (yes/no) was regressed on covariates found to be statistically different between SSI and non-SSI groups. The tolerable difference was pre-defined with a caliper of 0.2 per match [19]. Standardized differences were used to compare distributions before and after the matching process.

Datasets with missing or inconsistent cost data, or with typical procedures coded that indicate a SSI but with no corresponding ICD-code, have been excluded from the analysis.

All analyses were conducted using SPSS (version 19, IBM) and the R package for PSM.

## Results

### Prevalence of SSI

An SSI that was determined from the time of admission to discharge of the patient was reported in 4.9% (10,807/221,113) of the unadjusted study population. A significant difference of the SSI rate was associated between open and laparoscopic colorectal and thoracic procedures and between primary and revision orthopaedic procedures (Table 2). Of the 10,807 instances of SSI, 5,997 (55.5%) were classified as an A1/A2 infection and 4,810 (44.5%) as an A3 infection. A1/A2 infections were more prevalent for upper GI, colorectal and heart procedures, whereas A3 was the prevalent SSI for orthopaedic revision procedures. SSI frequency by procedure type and NRZ classification is shown in Table 2.

### Comparison of SSI and non-SSI groups

A comparison of the baseline characteristics and outcomes of the SSI and non-SSI groups for both unadjusted and adjusted analyses is shown in Table 3.

Age, sex, procedure type, immunosuppression, body mass index (BMI) ≥30 kg/m^2^ diabetes mellitus, Charlson comorbidity index (CCI) score (calculated via ICD-10 coding [20]) and preoperative length of stay were found to be statistically significant confounders for the occurrence of SSI and were adjusted for in the PSM.

**Fig 2:**
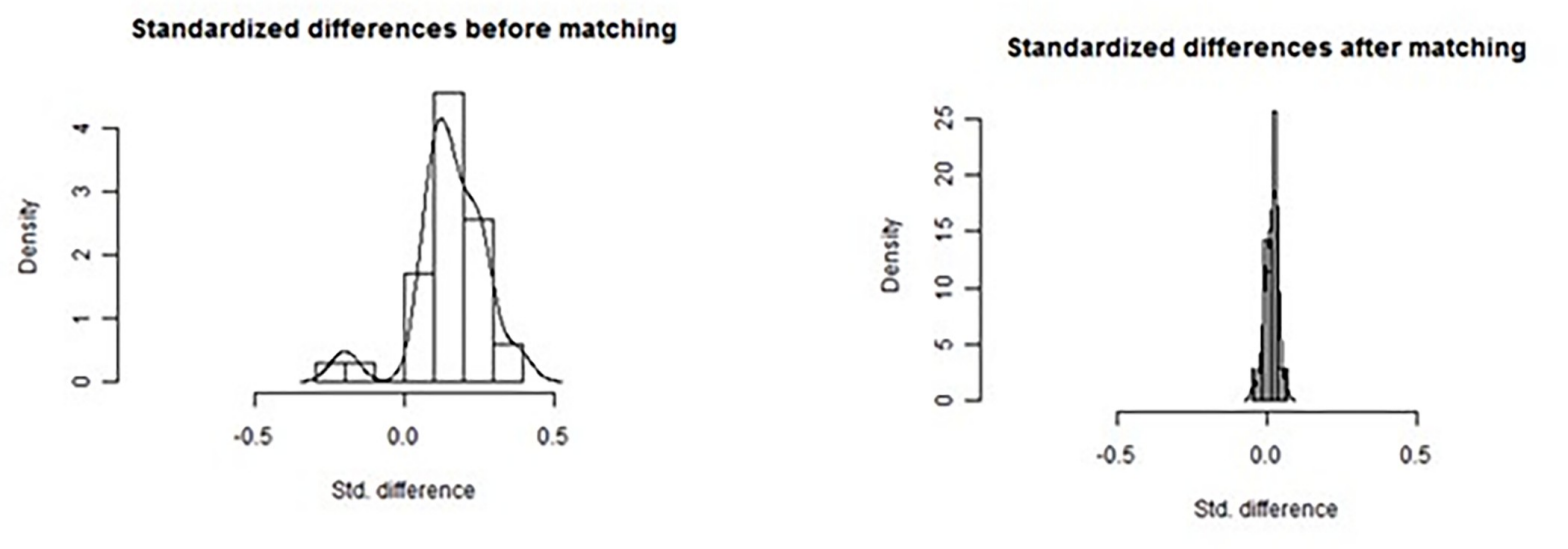
Cohort distribution before and after propensity score matching.

Following adjustment and 1:3 matching, the dataset consisted of 43,197 cases: 10,804 SSI and 32,393 non-SSI. The influence of the covariates was significantly reduced (Fig 2). In the matched cohort, no significant differences were found between SSI and non-SSI patients for age (67.4 years in both groups; p = 0.862), sex (55.6% female compared with 56.2% female; p = 0.428), diabetes mellitus (27.7% compared with 27.2%; p = 0.244), median CCI score (median [IQR]: 2 [4] compared with 2 [3]) and BMI ≥30 kg/m^2^ (16.7% compared with 16.9%; p = 0.634). However, the prevalence of immunosuppression (3.4% compared with 2.7%; p<0.001) was significantly higher for patients who had an SSI compared with those who did not. Procedure type and primary diagnosis were also significantly different between the groups (p<0.001).

In terms of hospital stay, patients who developed an SSI had a statistically significantly longer median overall and pre-surgical LOS than those that did not (median overall LOS: 28 days compared with 12 days [p<0.001]; median pre-surgical LOS: 2 days compared with 1 day [p<0.001]). Further, a statistically significantly higher proportion of patients with SSI was admitted to the ICU (55.2% compared with 30.9%; p<0.001). Mortality was also statistically significantly higher in the SSI group than in the non-SSI group (9.3% compared with 4.5%; p<0.001). The incidence of peritonitis (11.9% compared with 2.8%; p<0.001) and sepsis (17.7% compared with 3.3%; p<0.001) were also significantly higher in the SSI group.

Median case costs were statistically significantly higher for patients who had an SSI compared with those who did not (median [IQR]: € 19,008 [25,162] compared with €9,040 [7,376]; p<0.001). Also, the median reimbursement was significantly higher for the SSI group (€15,084 compared with €9,689; p<0.001). However, there was a negative median contribution margin for patients who had an SSI compared with a positive median contribution margin for non-SSI cases (-€1,534 compared with €633; p<0.001). From this it can be seen that SSI cases were under-reimbursed when compared with non-SSI cases.

**Fig 3:**
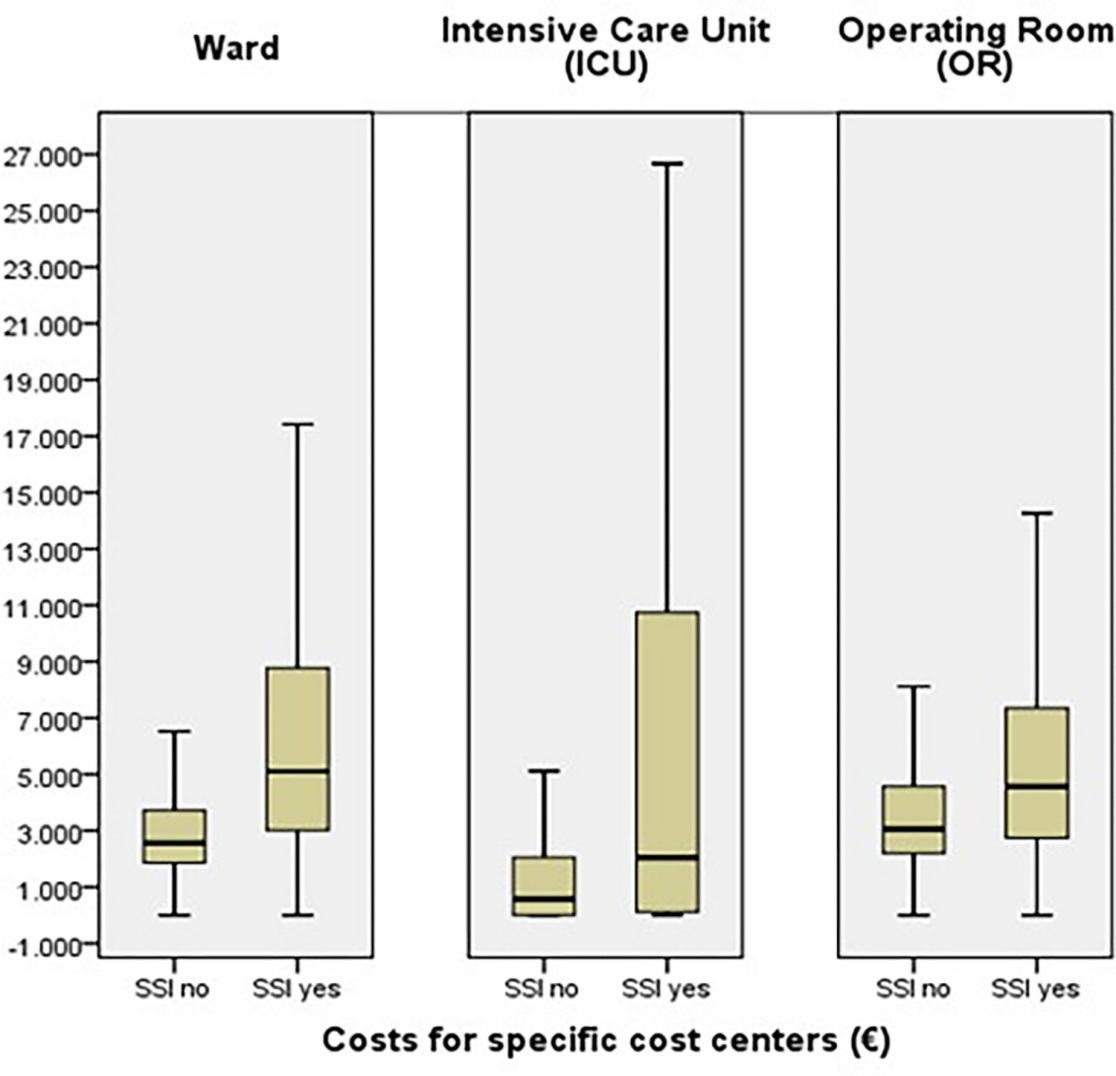
Median costs for SSI and non-SSI groups in specific cost centers.

Median costs for specific cost centers (ward, ICU and OR) were also statistically significantly higher for the SSI group than the non-SSI group (ward: €5,107 compared with €2,566 [p<0.001]; ICU: €2,014 compared with €568 [p<0.001]; OR: €4,564 compared with €3,059 [p<0.001]) but the variation was wide, particularly for ICU (Fig 3).

## Discussion

As one of the most common HAIs worldwide, SSIs require the attention of all disciplines involved in patient care, from health care professionals to hospital managers and payers [21].

This study is the first to analyze risk factors, prevalence, clinical consequences and economic burden of SSI in German hospitals from a single large dataset that is reflective of the actual hospital treatment required for the different types of SSI.

Due to their importance, SSI cases are coded to allow for calculation and compensation for the additional costs incurred. This increases the confidence in the dataset and ensures capture of a comprehensive range of variables. Previous publications have questioned the reliability of coded data [22]; however, measures implemented to ensure accurate capture have since improved in terms of reliability of coding within the G-DRG system.

The overall prevalence of in-hospital SSI in our study is considerably higher than that reported by KISS (4.9% compared with 1.08%). However, KISS is a voluntary reporting system which also contains post discharge SSI surveillance within 30 days and 90 days after the hospital stay and this highlights that the true SSI incidence is even higher than it has been previously thought to be [8]. Our study has also shown an increased proportion of SSI in male patients, which has been found in other clinical studies [23]. The KISS system is a well-designed and established surveillance system: so, the reasons for the differences in rates should be explored. SSI is a complication of a surgical procedure and is associated with a significant medical, and personal, burden for affected patients and their carers [4, 5, 21]. It is associated with a perceived negative view of the quality of surgery performed and potentially the reputation of the surgeons and hospital. Therefore, surveillance based on self-reporting and voluntary participation may be susceptible to under-reporting. By contrast, capture of data, for the purpose of reimbursement, is of interest to hospitals in order to receive remuneration for actual cost of surgical episodes. SSI surveillance, including the KISS system in Germany, shows the impact of SSI following different procedures in several countries [24]. A clinical trial conducted in the Netherlands is an example of this and supports the findings of our study, with an SSI prevalence of 2.2% for patients undergoing spine surgery (2.4% in the current study) [25].

Moreover, this study is exclusively based on in-hospital data and does not include information about post-discharge SSI. The total SSI rate of the investigated procedures may therefore have been even higher than the rates measured in this study [1, 26].

The prevalence of SSI in patients undergoing small bowel surgery in this study was 13.8%. This may be attributable to the broad variety of procedures in this group. It included patients undergoing pancreatic surgery with small bowel anastomosis, for which SSI rates of >10% are not unusual [27]. Due to low numbers in subgroups, analysis of this subset was deemed inappropriate. Slightly more SSIs were classified as A1/A2 than A3. Most SSI acquired following orthopaedic revision procedures were A3. This is expected due to the acknowledged high rate of prosthesis infection in revision surgery [28].

The impact of SSI on mortality has been repeatedly emphasized [29, 30]. The findings of the current study support those previously reported [4, 6]. This may be related to increased rates of sepsis and peritonitis, and the high rate of A3 infections. Future studies should focus more on this issue.

The results from this study also show that patients who develop SSI have, with 16 additional days, a significantly longer median LOS and incur much higher case costs for the hospital provider. Despite higher costs for the healthcare insurance, with additional reimbursement of €5,395 for cases with SSI, there is still an underfunding on the hospital site for each case with SSI. As many SSI are avoidable, these results create an incentive to fund preventive measures in order to avoid additional costs. The difference in contribution margin (reimbursement from healthcare insurance minus cost of the hospital) between SSI and non-SSI patients is €2,167. The G-DRG reimbursement does not consider the costs of occupied beds following an increased LOS. Accordingly, it is in the clinical, hospital, and social economic interest to avoid as many SSI as possible through appropriate infection prevention measures and the use of evidence-based SSI-reduction care bundles [31].

In terms of limitations, the retrospective and cross-sectional character of the study needs careful and cautious interpretation, as with all observational study types. Specifically, the distinction between superficial and deep incisional SSIs is limited because of the ICD coding system and CDC classification of SSI. A1, 2, 3 is not reflected 1:1 in the G-DRG coding system, as T81.4 is a broad and generic classification. We should have liked to have been more concise, based on the type of surgery, but the analysis we used was the only way we could analyze these large samples. Therefore, the approximations may result in some slight discrepancies than would be found using other global datasets [29]. The identification of SSI based on ICD-10 and translation to NRZ classification also has some limitations. Theoretically, T81.4 (‘infection following a procedure, not elsewhere classified’) could be coded as A1/A2 or A3. In addition, peritonitis is an infection which can be present on admission, or due to postoperative anastomotic leakage, and could represent an A3 SSI. To ensure that the identification and classification of peritonitis did not skew the results, it was reported as a co-morbidity as opposed to an SSI. However, this will have led to further under-reporting.

The majority of superficial incisional SSIs (A1/A2) are treated in primary care and not referred back to secondary care. As a result, no post-discharge follow-up data are available. Given the high percentage of up to 60% of SSIs occurring post discharge from hospital care [1, 26], the rate of SSI reported here is almost certainly an underestimate.

Furthermore, the measurement of the Charlson Comorditiy Score Index by ICD-10 Codes could lead to underestimating the score index due to errors in the coding (e.g. mild vs. moderate or severe liver disease).

Previous studies on the economic impact of SSI are mostly based on comparatively small or midsize sample sizes [4, 30]. Studies of healthcare-associated infection and costs on large patient populations are published for individual European countries. In a 2013 study there was an estimated average daily cost of €131 to €189 per day in private hospitals and of €166 to €304 per day in public hospitals for hospital care following a healthcare-associated infection, which was reported from France based on the evaluation of the compulsory hospital patient database PMSI (N = 520,715) [11]. A 2005 surveillance study in the UK, based on the Nosocomial Infection National Surveillance Service (NINSS) and an SSI dataset from 140 hospitals which included 67,410 patients, identified an extra LOS due to SSI which ranged from 3.3 days for abdominal hysterectomy to 21.0 days after limb amputation with an estimated additional ward cost of £290,60 per bed day due to SSI [6]. The dataset used for the present study comprises almost five million cases from across Germany, and with the analysis of the current study based on more than 220,000 cases, means its accuracy and generalizability is high. The data captured provide true hospital treatment costs of patients in Germany following the occurrence of SSI.

## Conclusion

This study represents the first to utilize comprehensive and robustly collected, “real world” data to demonstrate the clinical and economic burden of SSI in an inpatient setting across multiple clinical specialties in the German DRG-system. Despite limitations inherent to observational studies, this study offers what is considered to be an accurate reflection of clinical consequences and economic burden of SSI in hospitals in Germany. The prevalence of SSI in the examined data set, the statistically determined mortality and the significant underfunding of SSI cases calls for improvements in surveillance of SSI by using existing methods like NRZ. Pre-, intra- and postoperative efforts should be undertaken, with adequate compliance, to incorporate evidence-based SSI-reduction care bundles with high compliance to reduce the impact of this financially burdensome, and potentially fatal, postoperative complication.

## Supporting information

S1 Table Complete list of procedures of interest. (XLSX)

S2 Table Comparison of baseline characteristics and outcomes between SSI and non-SSI cohorts adjusted by a 1:1 propensity score matching. (XLSX)
